# Dielectric Walls/Layers Modulated 3D Periodically Structured SERS Chips: Design, Batch Fabrication, and Applications

**DOI:** 10.1002/advs.202200647

**Published:** 2022-03-24

**Authors:** Yi Tian, Haifeng Hu, Peipei Chen, Fengliang Dong, Hui Huang, Lihua Xu, Lanqin Yan, Zhiwei Song, Taoran Xu, Weiguo Chu

**Affiliations:** ^1^ Nanofabrication Laboratory CAS Key Laboratory for Nanophotonic Materials and Devices CAS Key Laboratory for Nanosystems and Hierarchical Fabrication CAS Center for Excellence in Nanoscience National Center for Nanoscience and Technology Beijing 100190 China; ^2^ Center of Materials Science and Optoelectronics Engineering University of Chinese Academy of Sciences Beijing 100049 China

**Keywords:** batch fabrication, dielectric walls/layers, localized surface plasmon resonance (LSPR), SERS chip design, surface plasmon polariton (SPP), trace detection

## Abstract

As an indispensable constituent of plasmonic materials/dielectrics for surface enhanced Raman scattering (SERS) effects, dielectrics play a key role in excitation and transmission of surface plasmons which however remain more elusive relative to plasmonic materials. Herein, different roles of vertical dielectric walls, and horizontal and vertical dielectric layers in SERS via 3D periodic plasmonic materials/dielectrics structures are studied. Surface plasmon polariton (SPP) interferences can be maximized within dielectric walls besieged by plasmonic layers at the wall thicknesses of integral multiple half‐SPP_plasmonic material‐dielectric_‐wavelength which effectively excites localized surface plasmon resonance to improve SERS effects by one order of magnitude compared to roughness and/or nanogaps only. The introduction of extra Au nanoparticles on thin dielectric layers can further enhance SERS effects only slightly. Thus, the designed Au/SiO_2_ based SERS chips show an enhancement factor of 8.9 × 10^10^, 265 times higher relative to the chips with far thinner SiO_2_ walls. As many as 1200 chips are batch fabricated for a 4 in wafer using cost‐effective nanoimprint lithography which can detect trace Hg ions as low as 1 ppt. This study demonstrates a complete generalized platform from design to low‐cost batch‐fabrication to applications for novel high performance SERS chips of any plasmonic materials/dielectrics.

## Graphical abstract

The roles of dielectrics in surface enhanced Raman scattering (SERS) via 3D periodic structures are recognized both theoretically and experimentally, which are quantitatively explored. The contribution from surface plasmon polariton interferences as secondary sources to SERS effects by designing the dimensions of dielectric walls can be one order of magnitude higher than that caused simply by roughness and/or nanogaps.

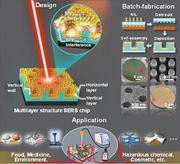

## 1 Introduction

Surface‐enhanced Raman scattering (SERS) is well recognized as a powerful spectroscopy technique for molecule detection in chemical, biological, environmental sciences, and other fields due to uniqueness of its vibrational fingerprints and high sensitivity.^[^ ^1^, ^2^, ^3^, ^4^ ^]^ Intrinsic performance of a SERS substrate is predominantly determined by electromagnetic field (EMF) enhancement, number, and distribution of so‐called hot spots which are normally referred to as specific locations of a structure and nanogaps formed by plasmonic materials/dielectrics.^[^ ^5^ ^]^ EMF enhancement at hot spots is strongly dependent on not only the local structures but also the effective optical properties of both plasmonic materials and dielectrics.^[^ ^5^, ^6^ ^]^

The reciprocal dependence of EMF enhancement on the size of nanogaps is well revealed in which the narrowing of nanogaps would enhance local surface plasmonic resonances (LSPR) and further EMF under the excitation of light.^[^ ^7^, ^8^ ^]^ The maximum EMF enhancement originating from the hot spots at small gaps for a plasmonic structure based on LSPR normally determines the SERS detection limit which is improved with the decrease of gap size (even down to 1 nm or less). This is the reason why the majority of SERS research attempts to construct the gaps of plasmonic structures as narrow as possible which is quite successful indeed. However, this requires more sophisticated fabrication tools and technologies. Also, the more formidable control of gap narrowing inevitably leads to the increase of the nonuniformity of EMF enhancements, which is unfavorable for the detection reproducibility of trace molecules.^[^ ^9^, ^10^ ^]^ Furthermore, such narrow gaps would make detection targets with certain sizes much more difficult to reach the hot spots, which even leads to the failure of detection. However, interfered SPP as a secondary excitation source to improve SERS effects can avoid the excessive pursuit of gap narrowing, and enhance the EMF at all hot spots in some particular zones significantly, without causing the additional nonuniformity of EMF enhancement. This undoubtedly benefits the fabrication of SERS chips and the detection of trace molecules, and thus their practical applications.^[^ ^9^, ^10^ ^]^ This reminds us of surface plasmon polariton (SPP) waves which can be generated to enhance EMF at all hot spots in some particular zones and thus more uniform SERS effects for specific plasmonic materials/dielectrics configurations such as 3D periodic structures under the excitation of light without reducing the nanogaps necessarily.^[^ ^11^, ^12^, ^13^ ^]^ Thus, design and creation of these specific 3D structures, especially dielectric configurations and dimensions (such as vertical walls) which may play an indispensable role in generation and interactions of SPP_plasmonic material‐dielectric_ (SPP_PM‐D_) waves, are particularly critical for achievement of strong SERS effects. Unfortunately, dielectrics received much less attention relative to plasmonic materials or were touched with focus simply on planar structures previously.^[^ ^14^, ^15^, ^16^ ^]^ The enhancement of SERS effects with respect to dielectrics could be understood in terms of Mie resonances or other mechanisms such as whispering‐gallery modes through the collection of both excited and inelastically scattered light.^[^ ^5^, ^6^ ^]^ The EMF enhancements based on different configurations of plasmonic and dielectric materials such as nanoparticles – on – mirror (NPoM), metal insulator metal (MIM) waveguide, and particles over dielectric surface were normally explored for horizontal dielectric layers.^[^ ^7^, ^14^, ^15^, ^16^ ^]^ Effective EMF enhancement can be realized for isolated nanoparticles and a metal film separated by a thin horizontal dielectric layer.^[^ ^15^ ^]^ The EMF enhancement decays against the increase of layer thickness exponentially with the corresponding SERS intensity which is quite sensitive to both the thickness and the dielectric constant.^[^ ^15^ ^]^ In reality, the EMF at the gaps for these structural configurations may be further enhanced through the coupling conversion between SPPs and LSPR via momentum matching due to their parallel electric fields (EFs).^[^ ^17^, ^18^, ^19^ ^]^

The multilayered horizontal planar structures above cannot provide an adequate platform for EMF and further SERS effects enhancement by virtue of SPP effects. However, 3D periodic structures composed of plasmonic materials/dielectrics (both horizontal and vertical) can be adopted as SERS chips to trap light, enhance both LSPR and SPP effects and their couplings, and strengthen the interactions between SERS chips and targets in a more effective way.^[^ ^16^, ^17^, ^20^, ^21^ ^]^ Furthermore, SERS structures by incorporating NPoM and/or MIM planar structures into the 3D periodic structures above are expected to show enhanced multiple coupling effects of LSPR and SPPs and thus effectively enhanced intrinsic EMF.^[^ ^22^ ^]^ The combination of sub‐nanometer gaps and micrometer‐scale resonators enables plasmon interference to multiply SERS signals compared with the plasmonic structures only.^[^ ^22^ ^]^ The propagations and behaviors of SPPs involved in the periodic dielectric structures as possible excitation sources of LSPR to remarkably enhance SERS effects were scarcely touched. Exploring the roles of dielectrics in 3D plasmonic structures can not only enrich the structures of 3D SERS chips but also create a novel platform for addressing fundamental issues about SPP and LSPR effects involved.

Herein, we designed novel 3D periodic Au/SiO_2_ SERS chips with intrinsically high performance through maximizing the coherent interferences of SPP_Au‐SiO_ **_2_** waves which were batch fabricated in wafer‐scale using conventional semiconductor processes of nanoimprint lithography (NIL) and self‐assembly.^[^ ^23^, ^24^, ^25^, ^26^, ^27^ ^]^ Within the 3D structures, different roles of SiO_2_ walls (vertical) and layers (vertical and horizontal) were explored. EF can be more effectively enhanced by optimizing the SiO_2_ walls thickness to maximize the interferences of SPP_Au‐SiO_ **_2_** waves other than the SiO_2_ layers (vertical and horizontal) via the coupling between SPP_Au‐SiO_ **_2_** and LSPR. This is beyond the conventional recognition. The SERS chips thus designed and fabricated were demonstrated to have excellent performances for detection of both Rhodamine 6G (R6G) model molecules and trace Hg ions in water. This study provides a complete generalized platform from design to wafer‐scale fabrication of novel SERS chips with intrinsically high performances, which would undoubtedly promote their practical applications.

## 2 Results and Discussion

**Figure 1:**
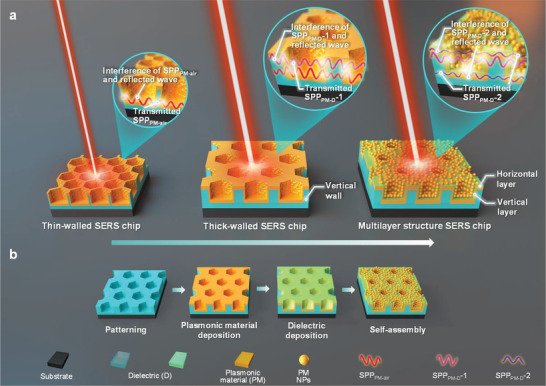
Schematic illustrations of SPPs interferences and their couplings with LSPR a) and preparation b) of two types of 3D periodic hollow hexagonal Au/SiO_2_ SERS chips, i.e., with vertical dielectric nanowalls only (Thick‐walled SERS chips) and extra vertical and horizontal dielectric layers (Multilayer structure SERS chips), respectively.

It is recognized that the remarkable SPP effects can be excited at both the Au‐air and Au‐SiO_2_ interfaces within a periodic structure by a polarized light in TM mode (i.e., The polarization direction of incident light is perpendicular to the periodic nanostructure.).^[^ ^20^, ^28^ ^]^ We established a generalized quantitative methodology of maximizing the SPP_Au‐air_ effects excited at the Au‐air interfaces to improve the SERS performance.^[^ ^20^, ^28^ ^]^ With standing wave effects, LSPR and SPP effects, and their couplings considered, excellent intrinsic SERS effects were achieved for the optimized periodic hollow hexagonal SiO_2_ frameworks deposited with a rough Au layer. The thickness, height, and opposite spacing of SiO_2_ framework walls is *T*_D_ = 9 nm, *H*_D_ = 198 nm, and *L*_D_ = 316 nm, respectively, and the nominal thickness of Au is 36 nm with a real thickness, *T*_PM_ ≈ 7 nm on the sidewalls,^[^ ^20^, ^21^ ^]^ as illustrated in the left panel of **Figure** **1**a (The thin‐walled structure). The dimension parameters of the periodic hollow hexagonal SERS structures are defined and shown in Figure S1 of the Supporting Information. The contribution of the maximized SPP effects to intrinsic SERS intensity by only optimizing the height and opposite spacing of dielectric SiO_2_ framework walls is about 144% that from LSPR caused simply by roughness and/or nanogaps, which is independent of roughness and nanogaps effects.^[^ ^20^ ^]^ However, the role of dielectric wall (vertical, the middle panel of Figure 1a) and layer thickness (vertical and horizontal, the right panel of Figure 1a) in 3D periodic metal/dielectric structures in SERS effects still remains elusive, which may greatly promote SERS effects through SPPs interferences and their couplings with LSPR. Herein, we designed and fabricated two types of 3D periodic Au/SiO_2_ SERS chips with vertical SiO_2_ walls, and vertical and horizontal SiO_2_ layers, respectively for exploring the role of dielectrics in intrinsic performance, as shown in Figure 1b.

### 2.1 3D Periodic Thick‐Walled (Vertical) Au/SiO_2_ SERS Chips with Rough Au Layers (Au RL/SiO_2_)

For a 632.8 nm laser, the wavelengths of SPPs excited at the Au‐air (*λ*_SPP Au‐air_) and Au‐SiO_2_ interfaces (*λ*_SPP Au‐SiO2_) are derived to be 603 and 388 nm, respectively.^[^ ^13^, ^28^ ^]^ SPP_Au‐air_ and SPP_Au‐SiO2_ (i.e., SPP_PM‐air_ and SPP_PM‐D_‐1) can interfere predominantly within the hexagonal air and SiO_2_ wall cavities besieged by Au sidewalls via Fabry–Pérot (FP) resonance‐like multiple reflections, respectively, as illustrated in Figure 1a; and Figure S1 of the Supporting Information. When the Au layer is thin (say, several nanometers only ^20^) enough, both SPP_Au‐air_ and SPP_Au‐SiO2_ waves could transmit the Au layer into the SiO_2_ and air cavities to be reflected by the opposite Au sidewalls, respectively, as shown in Figure 1. Without the transmission across the Au sidewall layers, the strongest interferences of SPP_Au‐air_ and SPP_Au‐SiO2_ with their reflected waves can be achieved for *L*_PM‐D_ ≈ 302 nm and *T*_D_ = 194 nm, i.e., integral multiple of half *λ*_SPP Au‐air_ and *λ*_SPP Au‐SiO2_, respectively.^[^ ^29^ ^]^ With the transmission of SPP_Au‐air_ waves across the Au layers to interfere within the SiO_2_ cavities, the coherent interference of SPP_Au‐air_ waves would take place at the SiO_2_ wall thickness of 206.5 nm, i.e., half the effective wavelength of 413 nm for SPP_Au‐air_ (*λ*_SPP Au‐air_/*n*_SiO2_ = 603 nm/1.461 = 413 nm with *n*_SiO2_ being the refractive index of SiO_2_ at 632.8 nm).^[^ ^20^, ^30^, ^31^ ^]^ Therefore, the maximum interferences of SPP_Au‐air_ and SPP_Au‐SiO2_ occurring at the Au‐air interfaces would be related to not only the SiO_2_ wall thickness but also the Au sidewall layer thickness which could act as secondary sources for excitation of extra LSPR and further remarkable enhancement of EMF. This can theoretically reveal the crucial role of dielectrics (Vertical walls) in enhancement of EMF and thus intrinsic SERS effects,^[^ ^17^, ^20^, ^21^, ^29^ ^]^ which will be further demonstrated in the following.

**Figure 2:**
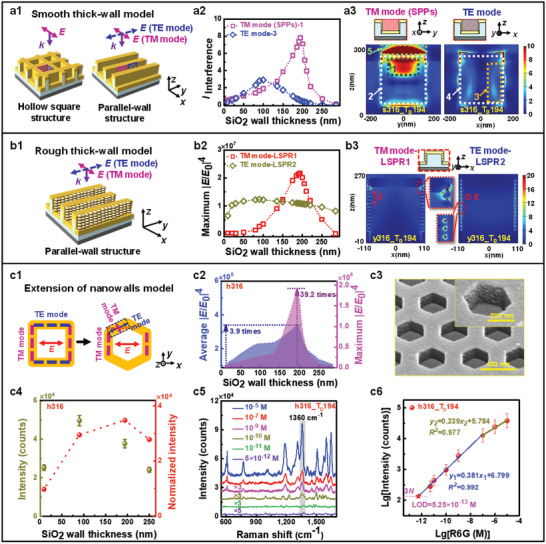
Design and calculations of 3D periodic hollow hexagonal Au RL /SiO_2_ SERS chips (h316_T_D_194) derived by combining the smooth hollow square s316_T_D_194 and parallel‐wall y316_T_D_194 models a) and the rough models b) and their experimental performance c). a1) Hollow square and parallel‐wall Au/SiO_2_ models with smooth thin Au layers. a2) SiO_2_ wall thickness dependences of maximum SPP (TM mode) and EM waves (TE mode) interference intensities at Regions “1” and “3”, respectively, calculated from the models in a1 using FDTD solutions. a3) Spatial distributions of |*E*/*E*_0_|^2^ based on s316_T_D_194 at TM and TE modes. b1) Parallel‐wall Au/SiO_2_ model with rough Au layers similar to that in a1). b2) and b3) Calculated SiO_2_ wall thickness dependences of maximum |*E*/*E*_0_|^4^ and spatial distributions of |*E*/*E*_0_| of the sidewalls at TM and TE modes based on the model in b1). c1) Extention from hollow square model with two pairs of parallel walls (s316) to hollow hexagonal model with three pairs of parallel walls (h316). c2) Calculated SiO_2_ wall thickness dependences of average and maximum |*E*/*E*_0_|^4^ for the SERS chips (228 nm high and 316 nm opposite‐wall spaced SiO_2_ hexagonal frameworks (h316) deposited by a nominal 36 nm thick Au layer). c3) Tilt SEM image of h316_T_D_194 SERS chips with an enlarged image. c4) Experimental and normalized SERS intensities of h316 chips decorated with 10^−5^ m R6G against SiO_2_ wall thicknesses. c5) Raman spectra of h316_T_D_194 decorated by R6G with the concentrations from 5.0 × 10^−12^ to 10^−5^ m. c6) The logarithmic relationship between the intensity of the 1360 cm^−1^ peak and the concentration for h316_T_D_194 derived from the intensities in c5).

In our previous work,^[^ ^20^ ^]^ SPPs interference intensities could be calculated in terms of the combination of quadrilateral and parallel‐wall models, which was successfully extended to other multilateral structures. Similarly, two models, i.e., hollow square and parallel‐wall Au/SiO_2_ structures with smooth Au layers and *H*_D_ = 228 nm derived from the maximum standing wave effects for a 632.8 nm wavelength shown in **Figure** **2**a1, can be adopted for calculations of the interference intensities of SPP and EM waves at the sidewall surfaces against the thicknesses of Au layers and SiO_2_ walls at both TM and TE modes,^[^ ^20^, ^32^ ^]^ as shown in Figure 2a2,a3; and Figures S2–S5 of the Supporting Information. For s(*L*_D_)_T_D_194 (hollow square) and y(*L*_D_)_T_D_194 (parallel walls along *y*‐direction) with a spacing of *L*_D_ = *L*_PM‐D_+2×*T*_PM_ (*L*_PM‐D_≈302 nm) and a SiO_2_ wall thickness of 194 nm, their average |*E*/*E*_0_|^2^ intensities (relative to the intensities of light waves) in Region “1” for TM mode decrease against the Au layer thickness, implying the gradually diminished transmission of SPP waves to the Au‐air interfaces as a consequence of the increased Au layer thicknesses, as shown in Figure 2a3; and Figures S2 and S3a of the Supporting Information. Implications of the abbreviations for the samples are presented in Table S1 of the Supporting Information. In contrast, the calculated interference intensities of SPP waves at Region “1” for s(*L*_D_)_T_D_194 (i. e. the ratios of average |*E*/*E*_0_|^2^ intensities for s(*L*_D_)_T_D_194 to those for y(*L*_D_)_T_D_194)^[^ ^20^ ^]^ could be maximized at the thickness of about 7 nm Au, i.e.*, T*_PM =_ 7 nm, as seen in Figure S3b of the Supporting Information. This could be attributed to the combined effects from the EMFs confinement and the SPPs interferences. The optimization of the Au sidewall layers thickness is required for both the effective transmission and strong interferences of SPPs waves which can then enhance SERS effects desirably. For the Au layers as thin as 7 nm and the SiO_2_ walls as thick as about 194 nm here, i.e., s316_T_D_194 with *L*_D_ = 302+2×7 = 316 nm, the SPP_Au‐SiO2_‐1 waves can undergo multiple reflections across the Au/SiO_2_ walls to generate the strongest interference intensity at the Au‐air interfaces with almost one order of magnitude higher relative to the incident light, as seen in Figure 2a2. This can also be seen from the calculated normalized (i.e., average) |*E*/*E*_0_|^2^ intensities at both Regions “1” (maximum intensity‐dominated) and “2” (average intensity‐determined) shown in Figures S3b and S5 of the Supporting Information. However, the interference intensities of EM waves excited at Regions “3” and “4” (maximum and average intensity‐determined, respectively) for the hollow square structure at TE mode peaked at the thickness of SiO_2_ walls of about 90 nm, much weaker than those of SPP_Au‐SiO2_‐1 waves, as shown in Figure 2a2; and Figure S5 of the Supporting Information. The contribution of SPP_Au‐air_ interference effects excited at the top smooth Au‐air interfaces shown in Region “5” to EF enhancement is quite little to be reasonably negligible due to the lack of hot spots (Figure 2a3). Therefore, it can be concluded that the geometry of 194 nm thick SiO_2_ walls, and 302 nm spacing of and about 7 nm thickness of Au layers enables the strongest interferences of both SPP_Au‐SiO2_‐1 and SPP_Au‐air_ waves to maximize the EMF enhancement.

The calculations above were performed on the hollow square and parallel‐wall structural models with smooth Au layers, as shown in Figure 2a. The corresponding parallel‐wall model with rough Au layers in Figure 2b1 was adopted for calculations of EF intensities at both TM and TE modes. Figure 2b2,b3; and Figure S6 of the Supporting Information show the SiO_2_ wall thickness dependences of maximum EF enhancements located at the topmost Au‐SiO_2_ sidewalls zones for y316_T_D_194 at TM mode (The EF of the incident light along the *x* direction) and at the height corresponding to one fourth of the incident wavelength (158 nm) for y316_T_D_90 at TE mode, which are dominated by the SPP _Au‐SiO2_‐1 interference enhancements (Figure S4, Supporting Information) and the standing wave effects of the incident light, respectively. The local EF directions at the gaps along the *z*‐direction at TM mode are actually determined by the *z*‐direction EF of the *x*‐direction‐propagating SPP interference waves as efficient secondary sources.^[^ ^28^ ^]^

Here, we can extend the similar analysis and calculations on the simple square model with only two pairs of sidewalls above (s316) to the hexagonal model (h316) with three pairs of parallel sidewalls by deconvoluting the incident light EF direction into the directions parallel (TE mode) and perpendicular (TM mode) to the parallel walls, as shown in the illustration of Figure 2c1.^[^ ^20^ ^]^ The total maximum and average |*E*/*E*_0_|^4^ against the SiO_2_ wall thickness for h316 were derived by summing up the maximum (Figure 2b2) and average (Figure S7, Supporting Information) |*E*/*E*_0_|^4^ from LSPR1 (TM mode) and LSPR2 (TE mode) multiplied by their respective coupling coefficients (Table S2, Supporting Information) which are related to the interference intensities of SPP or EM waves, and the angles between the walls and the polarization direction of the incident light, as shown in Figure 2c2. The maximum and average |*E*/*E*_0_|^4^ for h316_T_D_194 with optimum 194 nm thick SiO_2_ walls are about 40 and 4 times those for h316_T_D_9 with only 9 nm thick SiO_2_ walls, respectively. Undoubtedly, the maximum EF intensity for a SERS chip would determine its detection limit theoretically. For the increase of maximum |*E*/*E*_0_|^4^ by a factor of about 40, the contribution from LSPR and SPP effects is estimated to be 5.89 and 6.65 times, respectively, indicating the dominant contribution from the SPP effects.

The crucial role of dielectric SiO_2_ vertical walls in the periodic Au/SiO_2_ framework structures in SPP effects and EF enhancements has been well confirmed by calculations which can be further demonstrated experimentally. To this end, the SERS effects of 3D hollow hexagonal SiO_2_ frameworks deposited with a rough Au layer (Au RL/SiO_2_, the nominal thickness of Au is 36 nm with a real thickness, *T*_PM_ ≈ 7 nm on the sidewalls) by varying the thicknesses of SiO_2_ walls, i.e., *T*_D_ = 9, 90, 194, and 250 nm (denoted as h316_T_D_9, h316_T_D_90, h316_T_D_194, and h316_T_D_250, respectively) can be evaluated. These chips were fabricated according to the processes shown in Figure 1b, and their scanning electron microscopy (SEM) images are shown in Figure 2c3; and Figure S8 of the Supporting Information. Thickness dependences of the 1360 cm^−1^ peak intensity and their normalized ones for 10^−5^ m Rhodamine 6G (R6G) molecules decoration with a *x*‐polarized light are presented in Figure 2c4 for the assessment of their intrinsic performance. **^[^** ^27^ **^]^** Here, the normalized SERS intensities, as the intrinsic SERS intensity per hot spot, were derived from the experimental SERS intensities normalized by the corresponding ratios of sidewall (*S*_s_) to projection (*S*_p_) area for different structures. The normalized intensity first increases and then decreases with the SiO_2_ wall thickness, and the maximum intensity was achieved at 194 nm (i.e., *T*_D_ = 194), in good agreement with the calculations in Figure 2c2. The Raman spectra of R6G with different concentrations detected by h316_T_D_194 chips, along with their logarithmic relationships between intensity and concentration are shown in Figure 2c5,c6, respectively. The limit of detection (LOD) for R6G is 5.25 ×10^−13^ m which is decreased by about 50 times compared to 2.50 × 10^−11^ m for h316_T_D_9 chips (with an even higher number density of hot spots). So far, both calculations and experiments unambiguously demonstrated the unique and indispensable role of SiO_2_ vertical wall thickness in improvement of SPP wave interference effects, EMF enhancement, intrinsic performance and further detection limit of periodically structured SERS chips. Therefore, the thicker vertical SiO_2_ walls even with a lower density of hot spots can lead to the far better intrinsic performance compared to the thinner SiO_2_ walls with a sizably higher density of hots spots, indeed beyond the conventional recognition.

### 2.2 3D Multilayer Au/SiO_2_ SERS Chips by Introducing Extra Horizontal and Vertical Thin Dielectric Layers and Au Nanoparticles

**Figure 3:**
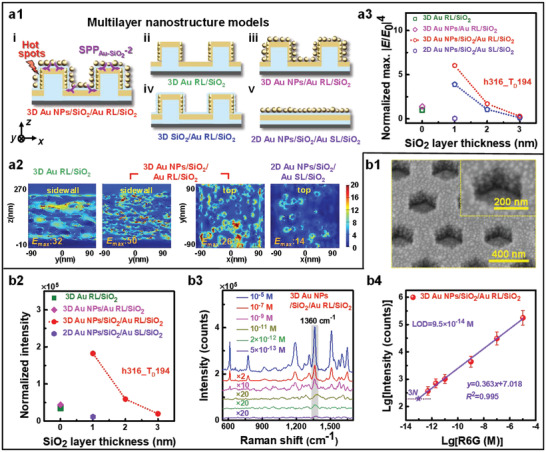
Structural schematics and EF calculations of diversified SERS chip models a) and morphology and experimental SERS results of 3D Au NPs/SiO_2_/Au RL/SiO_2_ chips (h316_T_D_194) b). a1) Five models with different multilayered SERS structures. a2) Spatial distributions of |*E*/*E*_0_| at surfaces calculated by FDTD solutions based on the models in a1). a3) Calculated SiO_2_ layer thickness dependences of the normalized maximum |*E*/*E*_0_|^4^ for the models in a1). b1) SEM images of 3D Au NPs/SiO_2_/Au RL/SiO_2_ h316_T_D_194 chips. b2) Normalized SERS intensities of different multilayered SERS structures. b3) Raman spectra of R6G with the concentrations from 5.0 × 10^−12^ to 10^−5^ m detected by h316_T_D_194 chips. b4) The logarithmic relationship between the peak intensity of 1360 cm^−1^ and concentration of R6G for h316_T_D_194 chips.

We have theoretically and experimentally established SiO_2_ wall (vertical) thickness dependences of SERS performance based on periodic hollow hexagonal Au RL/SiO_2_ chips above. To explore the roles of vertical and horizontal SiO_2_ layers, 3D Au NPs/SiO_2_/Au RL/SiO_2_ structures by depositing extra 1, 2, and 3 nm thick vertical and horizontal SiO_2_ layers followed by self‐assembly of Au nanoparticles based on Au RL/SiO_2_ were also studied both theoretically and experimentally, as shown in **Figure** **3**. For comparison, configuration‐modified models such as 3D Au NPs/Au RL/SiO_2_, 3D SiO_2_/Au RL/SiO_2_, and 2D Au NPs/SiO_2_/Au smooth layer (SL)/SiO_2_, along with their FDTD calculations are also given in Figure 3a1–a3; and Figures S9 and S10 of the Supporting Information. 3D Au NPs/SiO_2_/Au RL/SiO_2_ with 1 nm thick SiO_2_ layers was observed to show the strongest maximum |*E*/*E*_0_|^4^ at sidewall surfaces (Figure 3a2) normalized by that of 3D Au RL/SiO_2_ h316_T_D_194, and appears to have an exponential decay against the SiO_2_ layer thickness, as shown in Figure 3a3.^[^ ^14^ ^]^ The maximum |*E*/*E*_0_|^4^ for 3D Au NPs/SiO_2_/Au RL/SiO_2_ is about 6.0 times that of 3D Au RL/SiO_2_ with an increase by almost two orders of magnitude compared to 2D Au NPs/SiO_2_/Au SL/SiO_2_ (i.e., so‐called planar MIM structures in Figure 3a1). For 3D Au NPs/SiO_2_/Au RL/SiO_2_ with 194 nm thick walls, we further elaborated the calculations on the wall spacing dependences of the maximum |*E*/*E*_0_|^4^ using FDTD solutions, as shown in Figure S10 of the Supporting Information. A negligible difference of about a factor of 0.03 only was observed between designed *L*_D_ = 316 and *L*_D_ = 322 with the highest normalized intensity. Consequently, the introduction of extra horizontal ultrathin SiO_2_ layers and Au nanoparticles would not change the optimized dimensions of periodic 3D SERS framework structures and further the design roadmap.

Likewise, 3D Au NPs/SiO_2_/Au RL/SiO_2_, 3D Au NPs/Au RL/SiO_2_, and 2D Au NPs/SiO_2_/Au SL/SiO_2_ chips were also fabricated using the processes in Figure 1b for SERS tests, and their SEM images are shown in Figure 3b1; and Figure S11 of the Supporting Information. The 3D Au NPs/SiO_2_/Au RL/SiO_2_ chips with 1 nm thick SiO_2_ layers decorated by 10^–5^ m R6G show the normalized experimental intensities, about 5 times that of Au RL/SiO_2_ (h316_T_D_194, Figure 2c3) and about 16 times that of 2D Au NPs/SiO_2_/Au SL/SiO_2_, as shown in Figure 3b2. Therefore, the introduction of extra ultrathin 1 nm SiO_2_ layers and Au nanoparticles for h316_T_D_194 can further enhance the SERS effects.

So far, we preliminarily demonstrated both theoretically and experimentally the superior performance of structure‐optimized 3D Au NPs/SiO_2_/Au RL/SiO_2_ SERS chips. To further evaluate the performance of 3D Au NPs/SiO_2_/Au RL/SiO_2_ SERS chips in detail, R6G molecules with different concentrations were decorated for SERS tests, as shown in Figure 3b3,b4. A detection limit as low as 9.5 × 10^−14^ m, along with an SERS enhancement factor (SERS EF) as high as 8.9 × 10^10^ was successfully achieved and decreased by the factors of 263 and 6 compared to h316_T_D_9 and h316_T_D_194 chips, respectively, very pronounced among the ones reported on Au‐based SERS probes (Table S3, Supporting Information). Their detection uniformity was also assessed using 10^−7^ m R6G to show a relative standard deviation (RSD) as low as 5.13%, normally lower than those reported, as given in Figure S12 and Table S3 of the Supporting Information.

In brief, we discussed about the roles of dielectric layers and walls in three cases, i.e., thick SiO_2_ walls (vertical) for excitation and optimization of SPPs effects, so‐called MIMs with horizontal and vertical ultrathin SiO_2_ layers. The design of thick SiO_2_ walls to optimize the SPPs effects can enhance the EF and thus SERS effects most significantly which roughly gives an oscillating dependence as a result of multiple FP‐like resonances. The incorporation of MIM structures can further enhance the EF slightly which follows an intensity decay against the SiO_2_ layer thickness.^[^ ^7^, ^15^, ^17^, ^18^, ^19^ ^]^ On one side, the ultrathin vertical and horizontal dielectric spacers in MIM structures introduce small gaps and further induce extra hot spots (Figure 3a1).^[^ ^7^, ^15^ ^]^ On the other side, the SPPs excited via the top and bottom interfaces between Au and thin SiO_2_ layers (horizontal, SPP_Au‐SiO2_‐2, Figures 1a and 3a1) could also contribute to the EF enhancement by coupling with LSPR.^[^ ^17^, ^18^, ^19^ ^]^ This is totally different than the thick vertical SiO_2_ walls in which the EF intensity from the SPPs effects changes quasiperiodically against the wall thickness though both the EF enhancements originate from the SPPs effects. By comparing the maximum |*E*/*E*_0_|^4^ (i.e., |*E*_max_|^4^, *E*_0_ = 1) calculated by several models (see Figure 3a2,a3; and Figure S9 of the Supporting Information), the contributions to EF enhancements from different effects can be obtained. Comparing Models i, iii, and iv, vertical MIMs result in an increase of maximum |*E*/*E*_0_|^4^ by a factor of about 6.0 in which the introduction of the vertical thin dielectric layers with the thickness of 1 nm causes an increase by a factor of about 3.9 (i and iii), and Au NPs causes an enhancement of about 1.5 only (i and iv). Comparing Models i and v, the horizontal MIMs in 3D structures cause an about 5.5‐fold increase at the top (with the constructive interference of SPP_Au‐SiO2_‐2) and only an about 2.2‐fold increase at the bottom relative to the 2D MIMs. This shows that the vertical thick dielectric walls (SPP_Au‐SiO2_‐1 interference effects) have the largest contribution to EF enhancements, followed by the top horizontal thin dielectric layers (SPP_Au‐SiO2_‐2 interference effects), vertical thin dielectric layers (gaps), and Au NPs (only plasmonic structures) in sequence. This is of very important guidance for the design of high performance SERS chips.

For 3D Au NPs/SiO_2_/Au RL/SiO_2_ structure with 1 nm thick vertical and horizontal SiO_2_ layers, the maximum |*E*/*E*_0_|^4^ is located at the sidewall, about 14 times that at the top layer (75 times that at the bottom). When the thickness of SiO_2_ layers is increased to 2 nm, i.e., the increase of spacer between Au NPs and Au RL, the maximum |*E*/*E*_0_|^4^ at the sidewall decreases down to 28% (36.4^4^/50.0^4^). The maximum |*E*/*E*_0_|^4^ at the top changes little as the unchanged gaps between neighboring Au NPs (along *x*‐direction) and the weak influence of SPP_Au‐SiO2_‐2 interference effect by the thickness increase of the horizontal dielectric layers (see Figure 3a2; and Figure S9 of the Supporting Information). Therefore, the thin vertical dielectric layer plays a leading role in the maximum |*E*/*E*_0_|^4^ which is strongly dependent on the EMF effect at the sidewalls.

### 2.3 Construction of a Complete Generalized Platform from Design to Wafer‐Scale Fabrication to Applications of High Performance SERS Chips

The design concept of 3D periodic Au RL/SiO_2_ and its derived multilayered structures here can be extended to any other plasmonic materials/dielectrics systems. Here, hollow hexagonal bimetallic Ag–Au RL/SiO_2_ SERS chips were designed and fabricated to achieve the optimum performance following the design approach and the fabrication processes above from the perspective of the better comprehensive SERS performance of Ag–Au bimetals.^[^ ^21^ ^]^ For a 532 nm laser and about 7 nm thick Ag–Au sidewalls (alternate deposition of Ag and Au layers for 4 cycles followed by annealing), the opposite spacing *L*_D_ and the thickness of SiO_2_ walls *T*_D_ were designed to be about 268 nm (*L*_D_ ≈ *λ*_SPP Ag_‐_Au‐air_ / 2 + 14 = 508 / 2 + 14 = 268 nm) and 164 nm (*T*_D_ = *λ*_SPP Ag_‐_Au‐SiO2_ / 2 = 327 / 2 ≈ 164 nm), respectively, as shown in **Table** **1**; and Figure S13a of the Supporting Information. With the same height of 228 nm, h268_T_D_164 chips decorated with 10^−5^ m 4,4’‐Bipyridine (BPY) were employed for the successful detection of trace heavy metal Hg ions using a portable 532 nm laser Raman spectrometer.^[^ ^21^ ^]^ The schematics of detection, the SERS spectra for the concentrations of Hg ions from 5.0 ×10^−12^ (1 ppt) to 5.0 ×10^−5^ m (10 ppm) with an acquisition time of only 8 s, and the linear relationship (*R* ^2^ = 0.994) between intensity change and logarithmic concentration are shown in Figure S13b–S13d (Supporting Information). Trace Hg ions in water with a concentration as low as 4.19 ×10^−12^ m (about 0.84 ppt) were successfully detected which is about three orders of magnitude lower than 10 × 10^−9^ m, or 2000 ppt of the US threshold value for drinkable water.^[^ ^33^, ^34^, ^35^ ^]^ This reveals the strong detection power of hollow hexagonal bimetallic Ag–Au RL/SiO_2_ SERS chips designed predominantly from the perspective of SPP effects. The high performance of 3D hollow hexagonal bimetallic Ag–Au RL/SiO_2_ SERS chips designed and fabricated here enables on‐the‐spot rapid detection of trace pollutants using a portable or even hand‐held Raman spectrometer, which undoubtedly promotes their practical applications.

**Table 1:** The designed spacings and thicknesses of dielectric walls of hollow hexagonal structures according to the incident light wavelengths and optical properties of metals and dielectrics

Metal/Dielectric	*λ*_Laser_ [nm]	*n*_m_	*k*_m_	*n*_d_	*λ*_SPP metal ‐air_ [nm]	*λ*_SPP metal‐dielectric_ [nm]	*λ*_SPP metal ‐air_ / *n*_d_ [nm]	*L*_D_ [nm]	*T*_D_ [nm]
Au/SiO_2_	632.8	0.120^[^ ^30^ ^]^	3.300^[^ ^30^ ^]^	1.461^[^ ^31^ ^]^	603.0	388.3	412.7	316	194
Ag‐Au/SiO_2_	532	0.195^[^ ^21^ ^]^	3.372^[^ ^21^ ^]^	1.465	508.0	327.0	346.8	268	164
Au/NIL resist	632.8	0.120	3.300	1.642	603.0	334.2	367.2	316	167
Au/SiO_2_	785	0.080	4.6645	1.454	766.7	513.0	527.3	397	257

Designed dimensions of some Au and Ag–Au/dielectrics SERS chip structures for different laser wavelengths with a given Au thickness of about 7 nm are given in Table 1 and Table S4 of the Supporting Information based on the optical constants of some materials in Figure S14 of the Supporting Information. For hollow hexagonal Au/TiO_2_ SERS chips, the spatial distributions of EF intensities, and TiO_2_ wall thickness dependences of average and maximum |*E*/*E*_0_|^4^ are shown in Figures S15 and S16 of the Supporting Information, respectively. The peaks for the maximum |*E*/*E*_0_|^4^ appear at the optimized TiO_2_ thickness of 182 nm, due to the superposition of SPP_Au‐air_ (on the top layer) interference effects.

**Figure 4:**
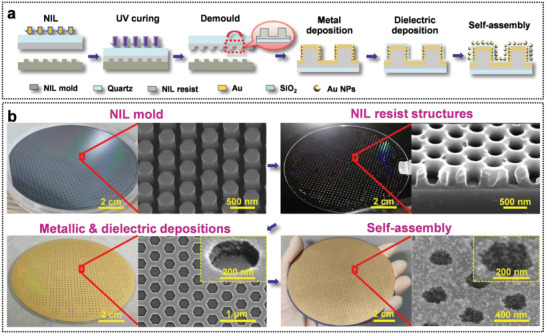
Batch‐fabrication processes of Au NPs/SiO_2_/Au RL/resist SERS chips. a) Diagram of fabrication processes of NIL with self‐assembly of Au nanoparticles. b) Photos and SEM images of a 4 in. NIL mold, nanoimprinted resist, metallic, and dielectric depositions and self‐assembly of Au nanoparticles structures.

The optimization of dielectric vertical wall thickness above can not only promote the EF enhancement of SERS chips significantly but also bring about great convenience of their batch fabrication due to the reduced aspect ratio of walls. With the substitution of resist for SiO_2_ frameworks in Au NPs/SiO_2_/Au RL/SiO_2_ chips, low‐cost and efficient nanoimprint lithography (NIL) instead of electron beam lithography (EBL) was employed for wafer‐scale fabrication of Au NPs/SiO_2_/Au RL/resist with a designed resist wall thickness of 167 nm, as shown in Table 1. We successfully fabricated more than 1200 SERS chips on a 4 in quartz substrate, as illustrated in **Figure** **4**. The real resist wall thickness is about 170 nm, slightly at variance with 167 nm as a result of multiple fabrication processes.

**Figure 5:**
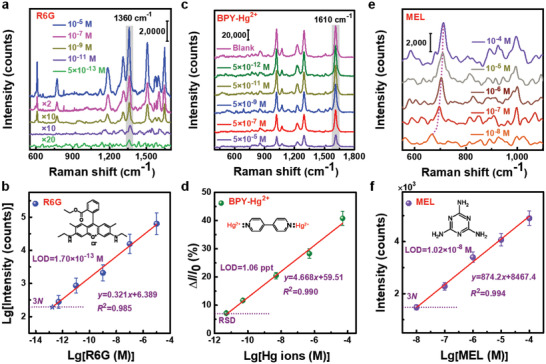
Applications of batch‐fabricated Au NPs/SiO_2_/Au RL/resist chips based on NIL to detection of R6G, Hg ions, and MEL. a,b) The Raman spectra of R6G with the concentrations from 5.0 ×10^−13^ to 10^−5^ m and the relationship between the logarithmic peak intensity of 1360 cm^−1^ and logarithmic concentration. c,d) The Raman spectra for Hg ions with the concentrations from 5.0 ×10^−12^ to 5.0 ×10^−5^ m and the relationship between the peak intensity variation of 1610 cm^−1^ and logarithmic Hg ions concentration. e,f) The Raman spectra of MEL with the concentrations from 10^−8^ to 10^−4^ m, and the relationship between the peak intensity of around 665 cm^−1^ and logarithmic concentration.

**Figure 6:**
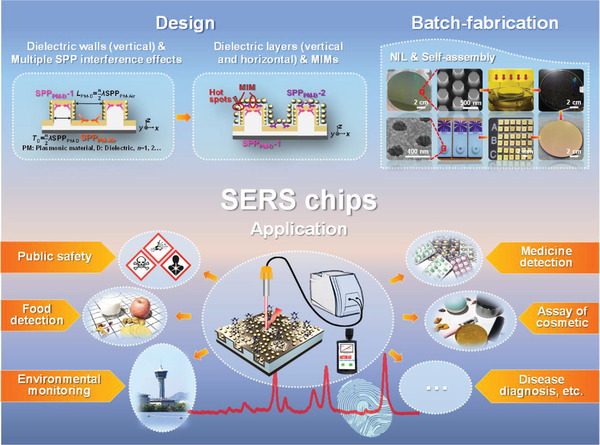
A complete generalized platform from design to wafer‐scale fabrication and applications of novel high performance SERS chips.

Au NPs/SiO_2_/Au RL/resist chips based on 4 in‐wafer were adopted for R6G detection in which a LOD as low as 1.7 × 10^−13^ m, an enhancement factor of 4.97 × 10^10^ and a nonuniformity of 6.95% (RSD for the chips on a 4 in. wafer) were achieved, as shown in **Figure** **5**; and Figure S17 of the Supporting Information. These chips were further applied to the detection of both heavy metal trace Hg ions in water and melamine (MEL) in alcohol with a LOD of 5.3 ×10^−12^ m (1.06 ppt, *R* ^2^ = 0.990) for Hg and a LOD of 1.02 × 10^−8^ m (*R* ^2^ = 0.994) for MEL using a 632.8 nm laser Raman spectrometer, which are also very outstanding among the literature,^[^ ^36^, ^37^, ^38^ ^]^ as shown in Figure 5. Therefore, the SERS chips designed by carefully considering the key role of dielectric vertical walls, and vertical as well as horizontal layers, and batch‐fabricated using the cost‐effective NIL processes show excellent detection performances for targets with different natures, which are of great promise in practical applications such as public security, environmental monitoring, health monitoring and disease diagnosis, and medicine, cosmetic, and food safety, as shown in **Figure** **6**.

## 3 Conclusion

A complete generalized platform for design, wafer‐scale fabrication, and facile applications of high performance 3D periodic SERS chips is well demonstrated. The crucial and indispensable roles of vertical dielectric walls in achievement of high performance of 3D periodic SERS chips were pioneeringly recognized both theoretically and experimentally in which usually thick dielectric walls enable the maximum FP‐resonance‐like interference effects of SPPs to further excite LSPR effects greatly as secondary sources. The incorporation of extra ultrathin horizontal and vertical dielectric layers (so‐called MIMs usually) in the SERS chips above can further enhance the performance in terms of SPPs effects as well but in a far less dominant and differing way. The SERS chips thus designed can be fabricated using not only expensive state‐of‐the‐art facilities such as EBL but also cost‐effective NIL processes with the successful achievement of 1200 chips for a 4 in wafer. Both the chips fabricated using two processes above show excellent detection performances for different targets. For typical R6G, the LOD of 9.5 × 10^−14^ m, the SERS EF of 8.9 × 10^10^ and the nonuniformity of 5.13% for EBL‐based chips, and the LOD of 1.7 × 10^−13^ m, the SERS EF 4.97 × 10^10^ and the nonuniformity of 6.95% for NIL‐based batch‐fabricated chips were realized. Both chips also realized the detection of trace Hg ions in water with the LOD as low as around 1 ppt and the chip with Au–Ag bimetallics as plasmonics could even realize the LOD as low as 0.84 ppt. The high performance of 3D periodic SERS chips designed and fabricated here enables on‐the‐spot rapid detection of trace pollutants using a portable or hand‐held Raman spectrometer, which undoubtedly promotes their practical applications, as well demonstrated. The platform proposed here can be generalized to the development of diversified novel 3D periodic plasmonics/dielectrics SERS chips with high performance, which would undoubtedly trigger considerations of novel effects and phenomena in SERS probe technology and accelerate their practical applications in environment, food and health, public security, and other fields.

## 4 Experimental Section

### Preparation of Au RL/SiO_2_ SERS Chips Based on EBL

3D periodic hollow hexagonal Au RL/SiO_2_ nanostructures were fabricated on Si/SiO_2_ substrates with 300 nm SiO_2_ thickness. First, the positive electron beam resist (ZEP‐520A) was spin coated on the sample and baked at 180 ℃ on a hot plate for 2 min. Then, hexagonal nanostructures were defined on the resist film based on the standard electron beam lithography (EBL, Vistec EBPG 5000+, Raith Company, Germany) and the following development process. The hollow nanostructures were fabricated by etching the 228 nm thick SiO_2_ layer using a mixture of CHF_3_ and Ar gases on an inductively coupled plasma etcher (SENTECH PTSA ICPRIE‐500, SENTECH Company, Germany) and then the residual resist was removed in an ultrasonic bath of butanone solvent. Finally, 1 nm thick Cr adhesion layers and Au films with the thicknesses of 36 nm were deposited using an electron‐beam evaporator (EBE, OHMIKER‐50B, Cello‐Tech Company, Taiwan, China). The fabricated structures were observed using a scanning electron microscope (NOVA NanoSEM 430, FEI Company, USA).

### Preparation of Au RL/Resist SERS Chips on 4 in. Quartz Substrate Based on NIL

First, the NIL resist (SuZhou Guangduo Micro, Nano‐Device Co., Ltd, China) with a thickness of about 300 nm was spin coated on a 4 in quartz substrate and baked at 100 ℃ on a hot plate for 1 min. The UVNIL was performed on Hex‐1 (Zwick/Roell Company, Germany). The nanoimprint pressure is 6000 N and hold time of UV exposure is 1500 s. Finally, 1 nm thick Cr adhesion layers and Au films with the thicknesses of 36 nm were deposited using EBE. The NIL mold with hexagonal Si nanopillars with 500 nm height was fabricated by EBL and an inductively coupled plasma etcher (Oxford Plasmalab System 100 ICP 180, Oxford Company, UK), and it was coated by a self‐assembled monolayer of 1H,1H,2H,2H‐perfluorooctyl trichlorosilane that reduce the surface energy to facilitate mold release.

### Preparation of Au NPs/SiO_2_/Au RL/SiO_2_ (or Resist) SERS Chips Based on Self‐Assembly

First, a thin SiO_2_ layer about 1 nm thick was deposited on the Au RL/SiO_2_ (or resist) SERS substrates fabricated based on EBL or NIL by a plasma enhanced atomic layer deposition equipment (PE‐ALD, SI, SENTECH Instruments GmbH, Germany) with the Bis (diethyl amino) silane (BDEAS) precursor and oxygen plasma gases. The growth temperature was kept at 200 ℃ and the deposition rate was about 0.056 nm per cycle. The optical constants and thicknesses of the SiO_2_ films were determined using a spectroscopic ellipsometer (SE 850 DUV, Sentech Company, Germany). Then, these SiO_2_/Au RL/SiO_2_ (or resist) substrates were immersed in ethanol solution of aminopropyltrimethoxysilane (APTMS, volume ratio of 5%) at 22 ℃ for 24 h and were rinsed with a copious amount of ethanol to remove extensive absorbed APTMS. After this, the modified substrates were immersed in Au NPs colloids at 37 ℃ for 20 h and were rinsed with DI water. Au NPs colloids were purchased from BBI Solutions (UK). After self‐assembly, these substrates were exposed to UV light for 5 min to decompose APTMS, then rinsed with ethanol and DI water, and dried.

### FDTD Calculations

Finite‐difference time‐domain (FDTD) method was used to calculate the spatial distributions of the electromagnetic fields. For simplicity, the Au RL/SiO_2_ models were used with periodically arranged hemisphere‐like Au nanoparticles with diameter of 13 nm on the sidewalls of SiO_2_ nanowalls in which the sizes of Au particles were basically derived from SEM observations. Periodic boundary conditions for the *xz* and *yz* planes were applied to simulate an infinite array of periodic nanostructures. Perfectly matched layer (PML) boundary conditions were used in the *z*‐direction. The mesh size used in the simulation region was 0.75 nm.

### SERS Detection

Some SERS measurements were performed using a 632.8 nm laser with a power of 1 mW and the *x*‐polarization on a Raman spectroscopy (Renishaw inVia, Renishaw company, UK) equipped with a 50× objective (NA = 0.5) and an integration time of 10 s. Others were performed using a 532 nm laser with a power of 30 mW on a portable Raman spectrometer (i‐Raman plus, B&W TEK INC., USA) equipped with a 40× objective (NA = 0.65) and an integration time of 8 s. For detections of R6G and MEL, the samples were first immersed into R6G aqueous solution and MEL absolute ethanol solution respectively for 12 h, and then dried naturally in air as SERS chips for molecules detections. For detection of Hg ions, the samples were first immersed into BPY absolute ethanol solution with a concentration of 10^−5^ m for 4 h, and then dried naturally in air as SERS chips for Hg ions detection. 35 µL of Hg ion solutions with different concentrations were dropped onto the SERS chips, respectively, then kept for 10 min, and finally dried in air. Likewise, 35 µL of deionized water was prepared with the same procedure as SERS chips for the blank control group. For each sample, measurements on at least five different positions were taken.

## Conflict of Interest

The authors declare no conflict of interest.

## Supporting information

Supporting information

## Data Availability

The data that support the findings of this study are available from the corresponding author upon reasonable request.
