# Supplementary material for: Dielectric Walls/Layers Modulated 3D Periodically Structured SERS Chips: Design, Batch Fabrication, and Applications
Source: Adv Sci (Weinh). 2022 Mar 24;9(15):2200647. doi: 10.1002/advs.202200647 (PMC9130881; doi:10.1002/advs.202200647)
Supplement: Supplementary file 1 — Supporting information [file ADVS-9-2200647-s001.pdf]

ADVANCED SCIENCE
Open Access

Supporting Information

for *Adv. Sci.*, DOI 10.1002/advs.202200647

Dielectric Walls/Layers Modulated 3D Periodically Structured SERS Chips: Design, Batch Fabrication, and Applications

Yi Tian, Haifeng Hu, Peipei Chen, Fengliang Dong, Hui Huang, Lihua Xu, Lanqin Yan, Zhiwei Song, Taoran Xu and Weiguo Chu**

Supporting Information

Dielectric Walls/Layers Modulated 3D Periodically Structured SERS Chips: Design, Batch Fabrication and Applications

Yi Tian, Haifeng Hu, Peipei Chen, Fengliang Dong, Hui Huang, Lihua Xu, Lanqin Yan, Zhiwei Song, Taoran Xu, and Weiguo**

Dr. Y. Tian, Dr. H. Hu, Prof. P. Chen, Prof. F. Dong, H. Huang, L. Xu, L. Yan, Z. Song, T. Xu, Prof. W. Chu
Nanofabrication Laboratory, CAS Key Laboratory for Nanophotonic Materials and Devices, CAS Key Laboratory for Nanosystems and Hierarchical Fabrication, CAS Center for Excellence in Nanoscience, National Center for Nanoscience and Technology, Beijing 100190, China.
E-mail: wgchu@nanoctr.cn; chenpp@nanoctr.cn
Prof. P. Chen, Prof. F. Dong, T. Xu, Prof. W. Chu
Center of Materials Science and Optoelectronics Engineering, University of Chinese Academy of Sciences, Beijing 100049, China.
E-mail: wgchu@nanoctr.cn; chenpp@nanoctr.cn
These authors contributed equally: Yi Tian, Haifeng Hu.

Supplementary Figures

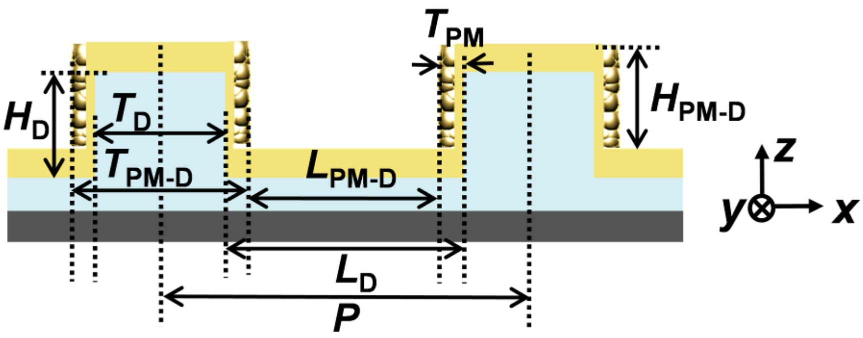

Figure S1. The lateral view and dimension parameters of periodic hollow hexagonal Au rough layer (RL) / SiO_2 nanostructures. Spacing, thickness, height and center distance of

SiO_2 nanowalls L_D, T_D, H_D and P, respectively, spacing, thickness and height of Au RL/SiO_2 nanowalls $L_{PM\text{-}D}$, $T_{PM\text{-}D}$ and $H_{PM\text{-}D}$, and thickness of Au RL at sidewall T_{PM} respectively, are defined in the schematic.

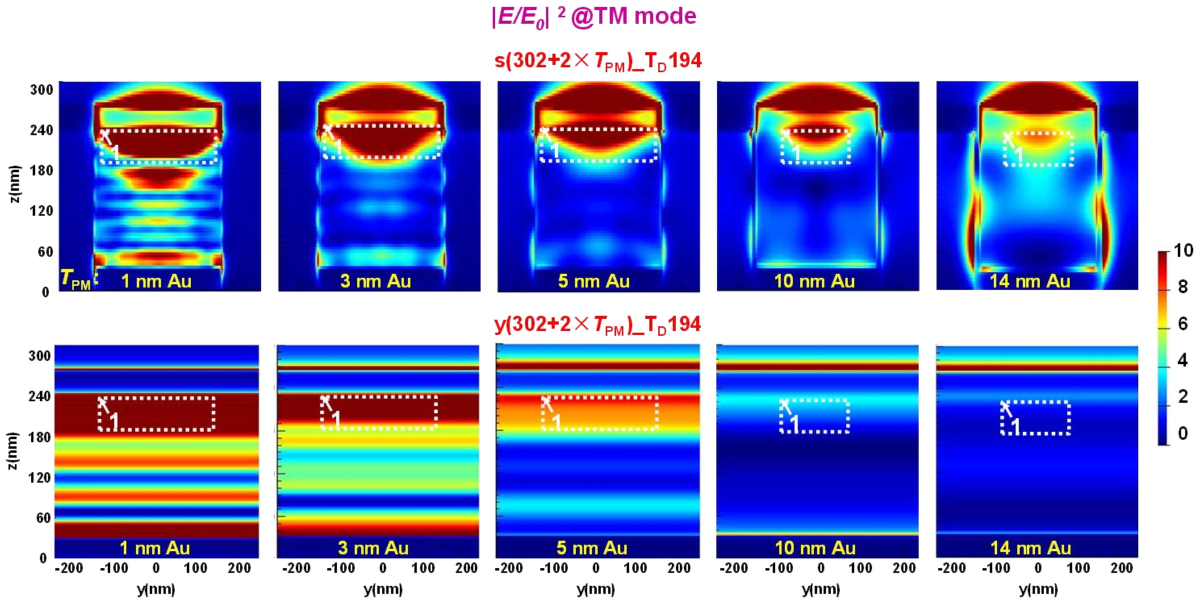

Figure S2. The spatial distributions of calculative $|E/E_0|^2$ intensities at the sidewalls of hollow square structure and y-nanowall arrays with different thicknesses of lateral Au layers at TM mode.

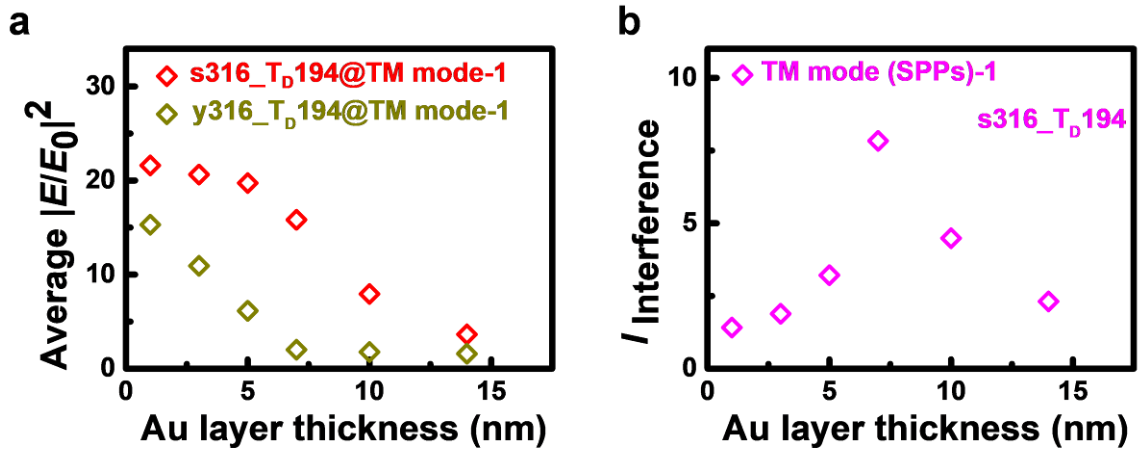

Figure S3. Calculations of average $|E/E_0|^2$ and SPP waves interference intensities at the sidewalls of hollow square and y-nanowall array structures with different lateral Au layers thicknesses at TM mode. a, The average $|E/E_0|^2$ at "1" regions of the sidewalls of s316_T_D194 and y316_T_D194 with different lateral Au layers thicknesses. **b,** The calculated

SPP waves interference intensities at "1" regions of the sidewalls of s316_T$_D$194 with different lateral Au layers thicknesses.

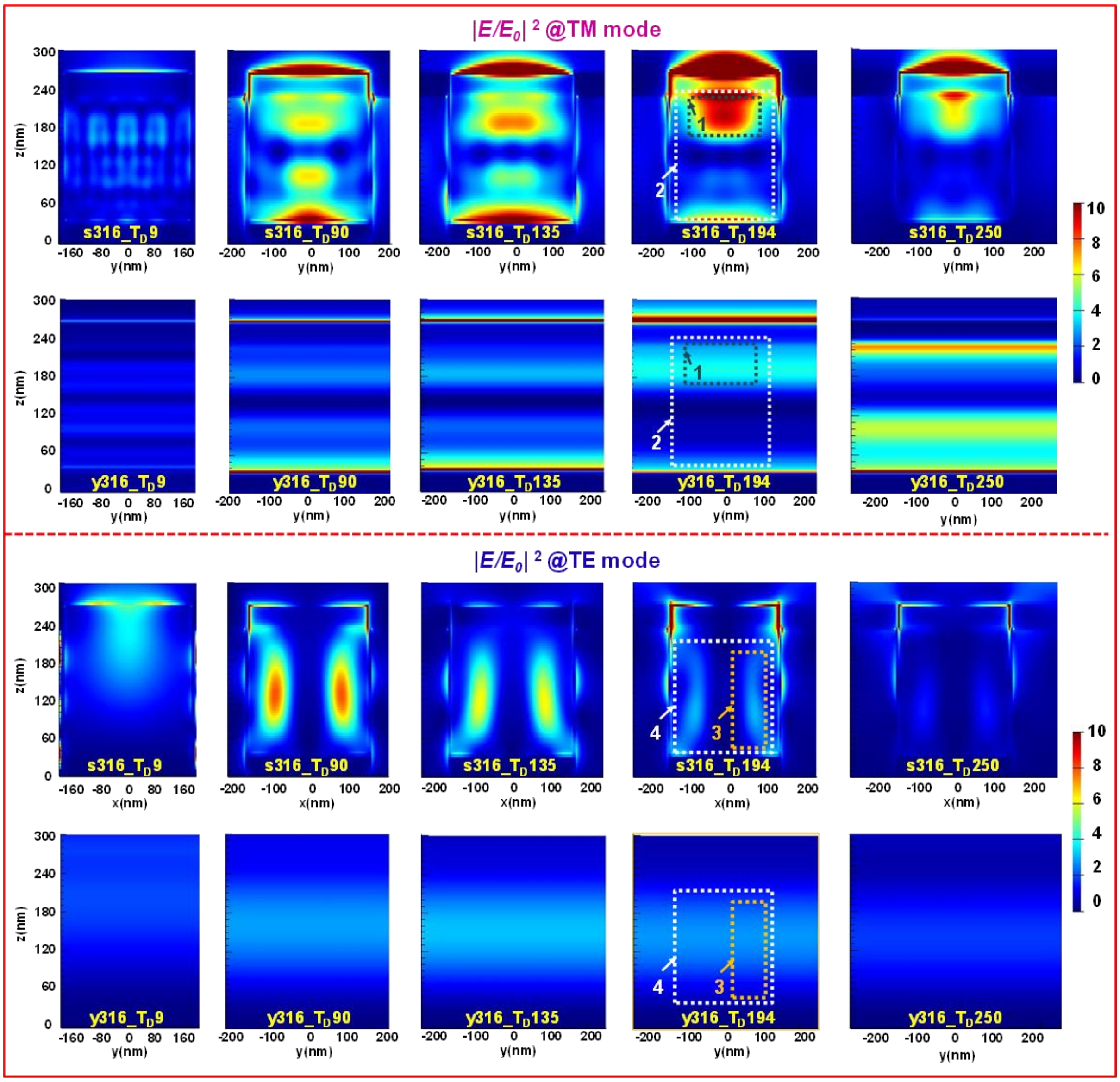

Figure S4. The spatial distributions of calculative $|E/E_0|^2$ intensities at the two kinds of sidewalls (TM and TE mode) of hollow square structures and y-nanowall arrays with 302 nm Au-Au sidewalls, 7 nm lateral Au layer and different SiO_2 wall thicknesses.

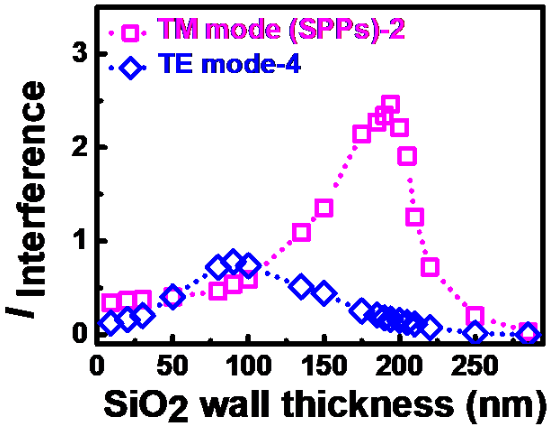

Figure S5. **Calculations of SPP and EM waves interference intensities at "2" and "4" regions of sidewalls surfaces (corresponding to TM and TE mode) of hollow square structures with 302 nm Au-Au sidewalls, 7 nm lateral Au layer and different SiO_2 wall thicknesses.**

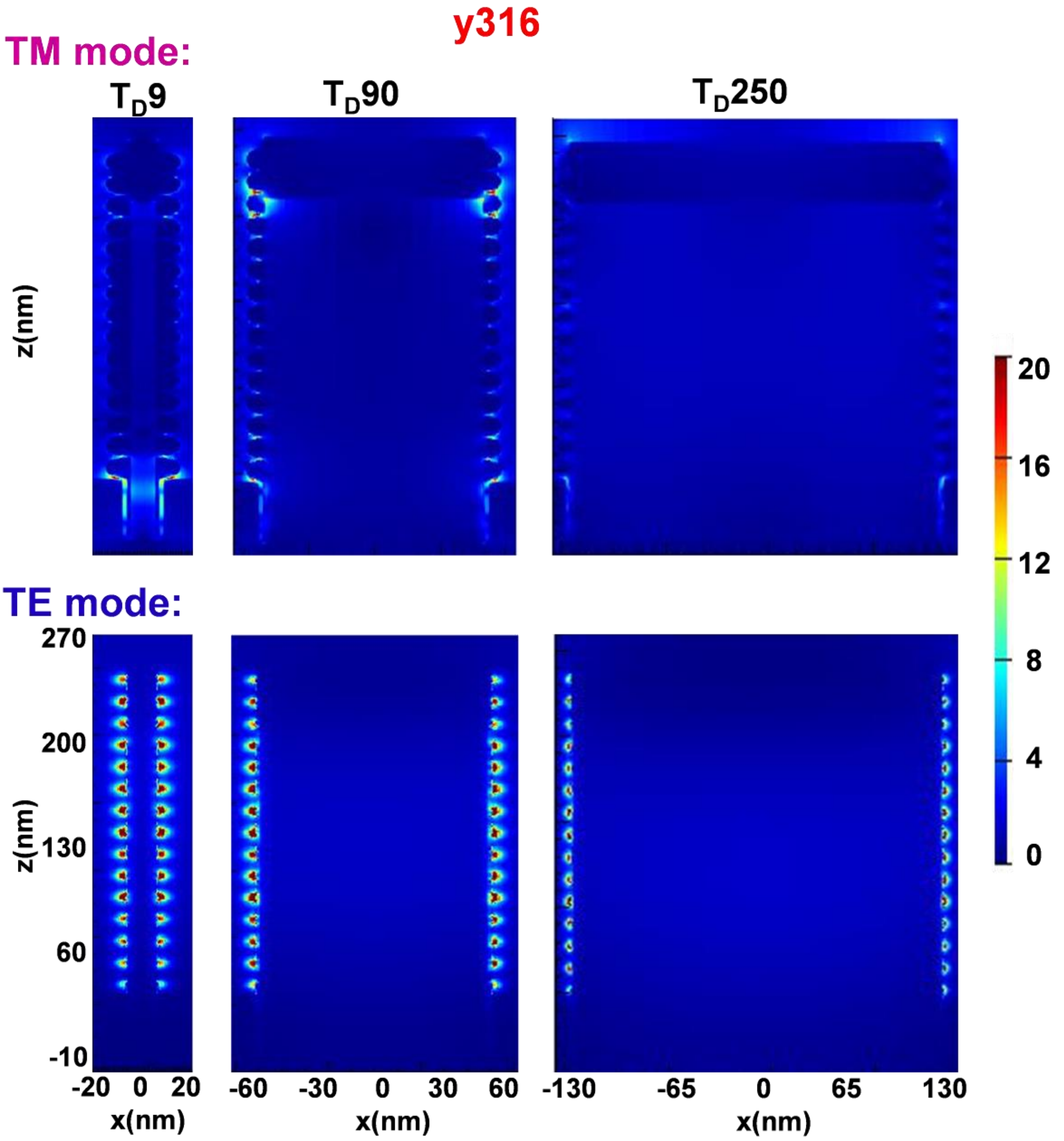

Figure S6. The calculative EF intensities at the sidewalls of rough y-nanowall arrays with different SiO_2 wall thickness at TM and TE mode, respectively.

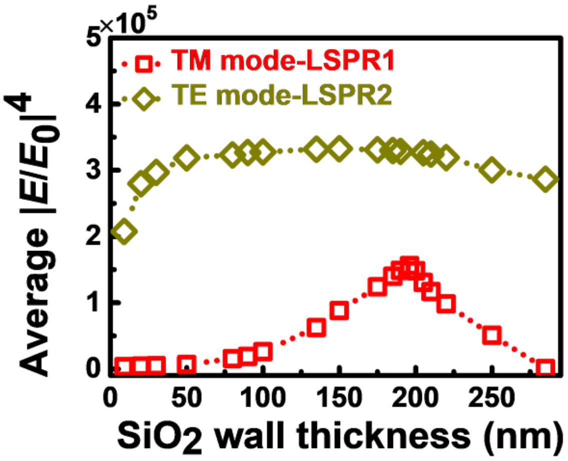

Figure S7. The calculative average $|E/E_0|^4$ at the sidewalls of rough y-nanowall arrays with different SiO_2 wall thickness at TM and TE mode, respectively.

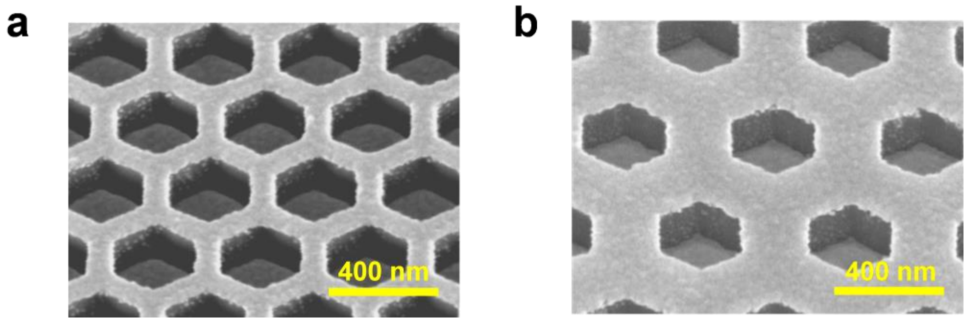

Figure S8. SEM images of 3D periodic hollow hexagonal 36 nm Au/ 228 nm SiO_2 SERS chips. a, and b, SEM images of h316_T_D90 and h316_T_D250, respectively.

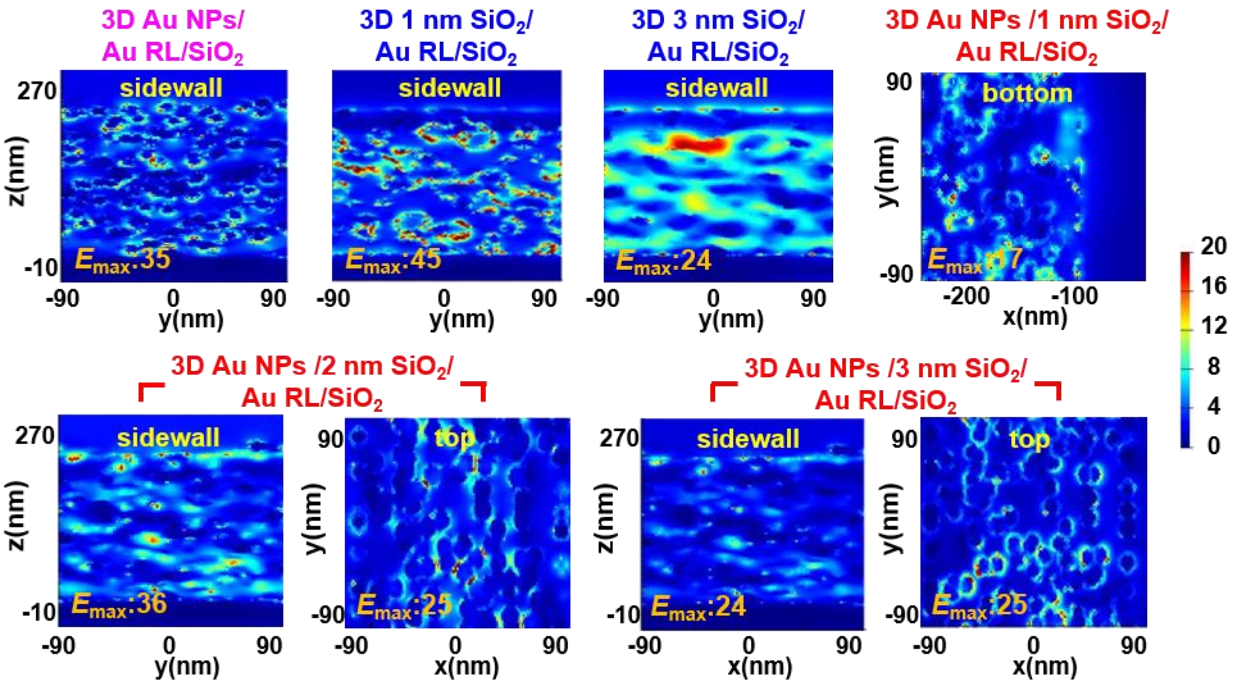

Figure S9. Calculated spatial distributions of $|E/E_0|$ at the sidewalls for 3D Au NPs/Au RL /SiO_2, 3D SiO_2/Au RL/SiO_2 with 1 and 3 nm thick SiO_2 layers and at the bottom, sidewall and top surfaces for 3D Au NPs/SiO_2/Au RL/SiO_2 h316_T_D194 multilayer structures with 1, 2 and 3 nm thick SiO_2 layers.

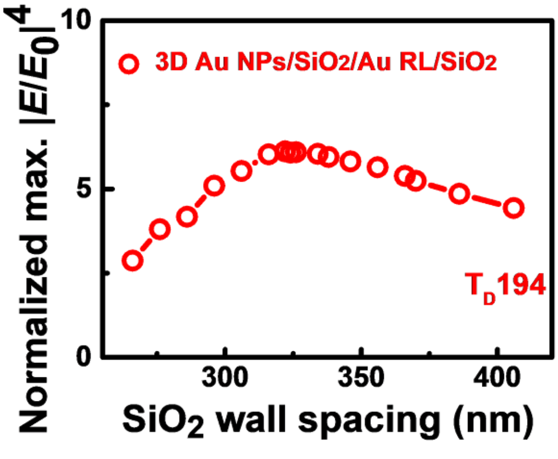

Figure S10. The calculations of SiO_2 wall spacing dependences of the maximum $|E/E_0|^4$ using FDTD solutions for 3D Au NPs/SiO_2/Au RL/SiO_2 with the wall thickness of 194 nm.

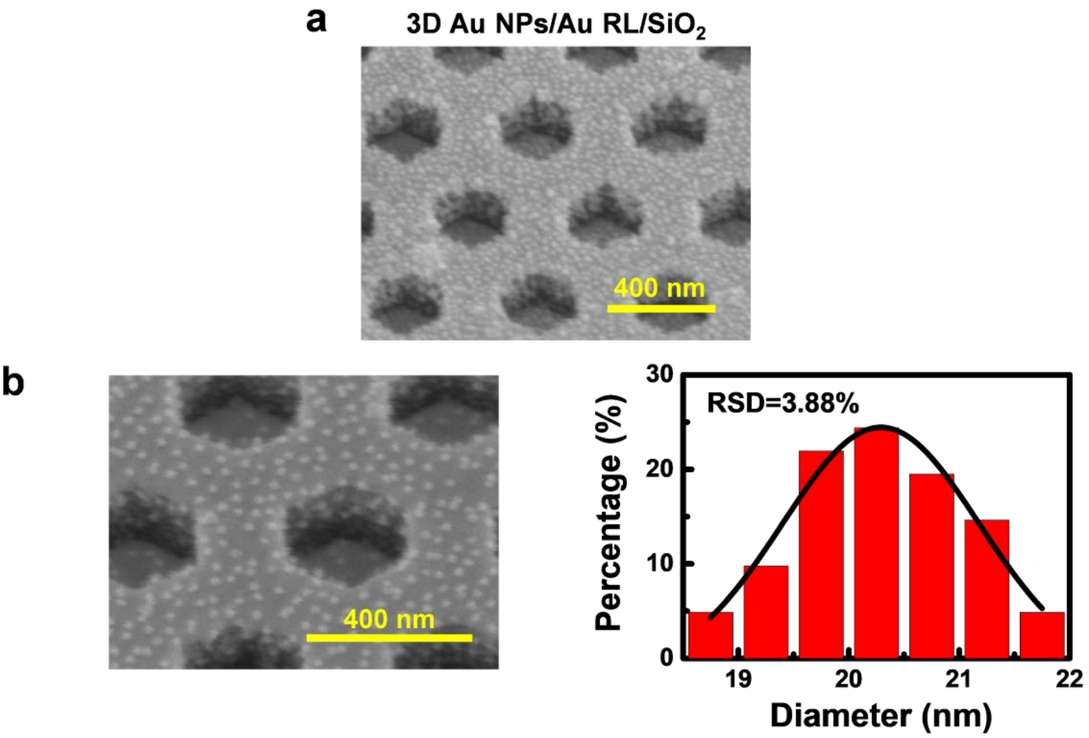

Figure S11. SEM images, Au NPs size distribution of Au/SiO_2 multilayer nanostructure. **a,** SEM image of 3D Au NPs/Au RL/SiO_2 nanostructures with Au particles of about 10 optical density. **b**, The size distribution of Au NPs coming from the purchase for self-assembly, based on SEM images. The average diameter of Au NPs is 20.2 nm.

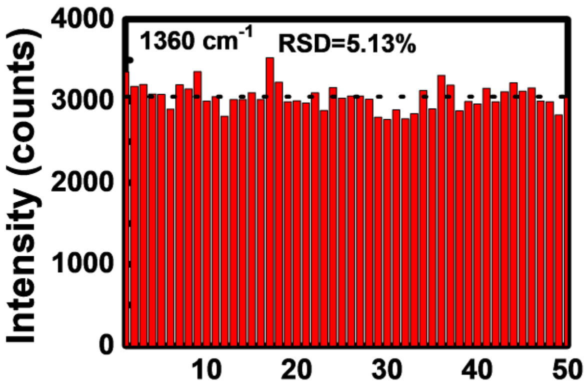

Figure S12. SERS performance of 3D Au NPs/SiO_2/Au RL/SiO_2 h316_T_D194 chip with 1 nm thick SiO_2 layer. The intensity distribution of the peaks at 1360 cm^{-1} of the 10^{-7} M R6G -decorated this kinds of SERS chip with the corresponding RSD of 5.13%.

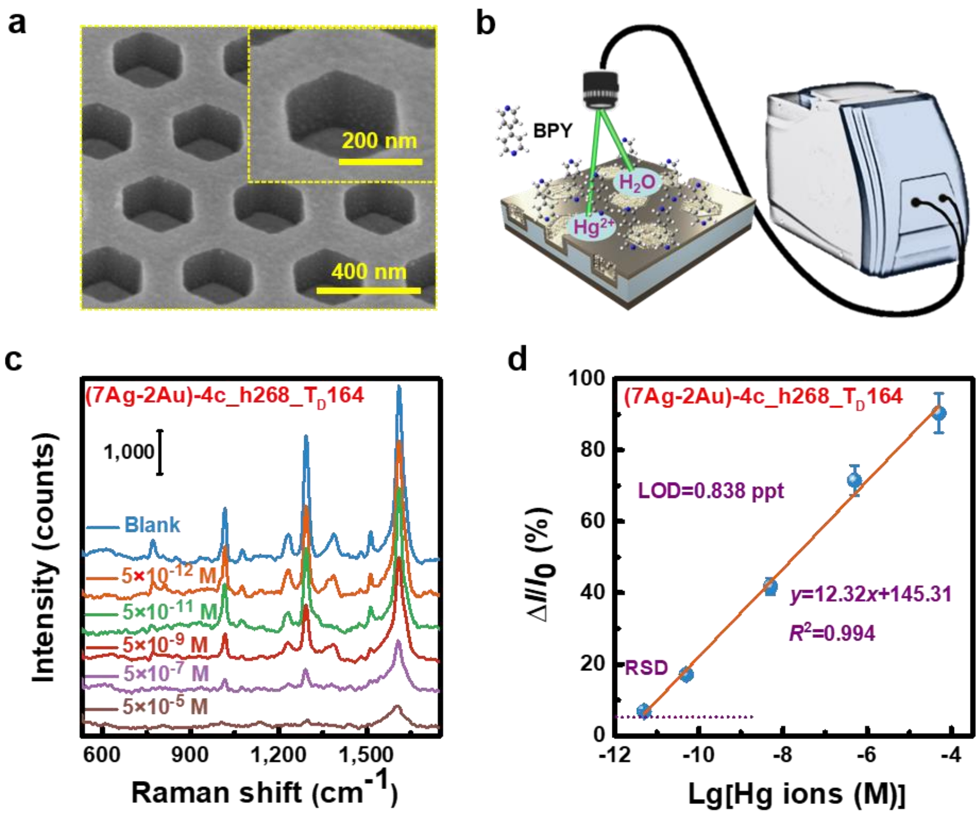

Figure S13. The design and application of optimized hollow hexagonal Ag-Au RL/SiO_2 SERS chips (h268_T_D164). a The SEM images of the annealed (7 nm Ag-2 nm Au)-4c/228 nm SiO_2_h268_T_D164 SERS chips. **b** The schematic diagram of Raman detection using a portable Raman spectrometer with 532 nm laser. **c** The Raman spectra of BPY detected Hg ions of 5.0 $\times 10^{-12}$ to 5.0 $\times 10^{-5}$ M. **d** The relationship between intensity variation and the concentration of Hg ions.

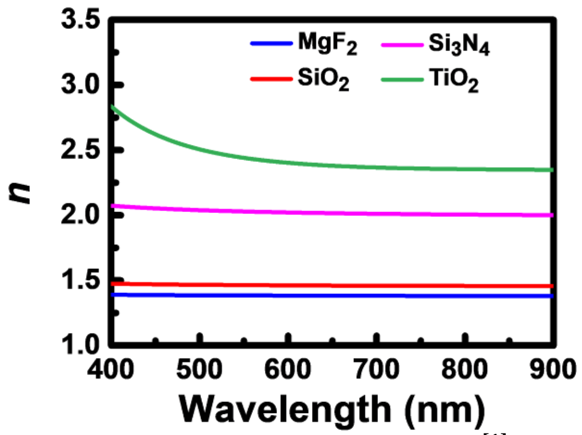

Figure S14. Optical constants of several dielectric, MgF_2[1], SiO_2[2], Si_3N_4[1], and TiO_2[3].

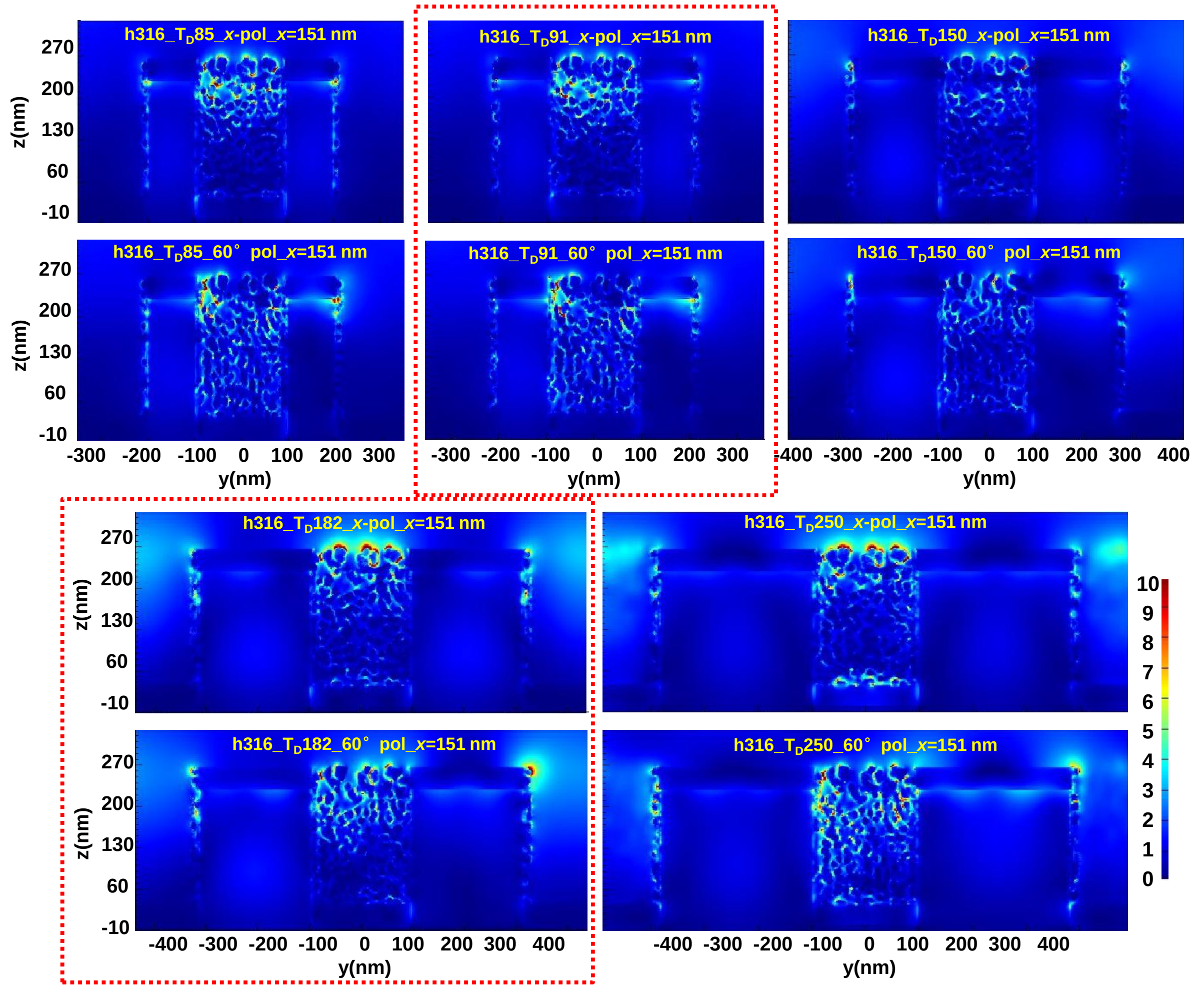

Figure S15. The spatial distributions of EF intensities for two kinds of nanowalls with different thicknesses from hollow hexagonal Au RL/TiO_2 nanostructures in *x*-polarized light, using the model with gold particle of random distribution.

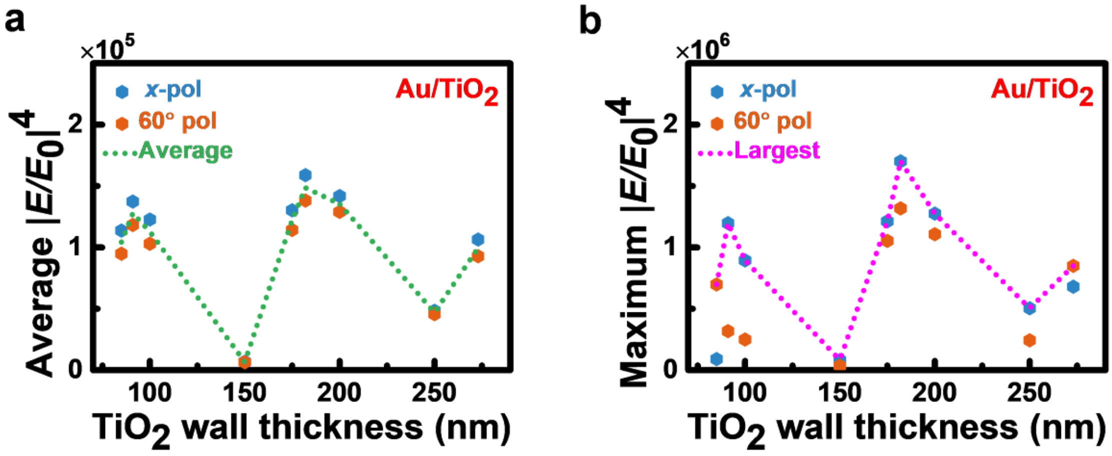

Figure S16. TiO_2 nanowall thickness dependences of average (a) and maximum (b) $|E/E_0|^4$ for h316 from hollow hexagonal Au RL/TiO_2 nanostructures in *x*-polarized light using the model with gold particle of random distribution. Based on the FP-resonance-like interference effects of SPPs, the interference intensity of $SPP_{Au\text{-}TiO2}$-1 changes periodically with the increases of TiO_2 wall thickness. The coupling of $SPP_{Au\text{-}TiO2}$-1 and $SPP_{Au\text{-}air}$-2 (on the top layer) results in the maximum EF enhancement locating in the hot spots at the siedwall near the top with 182 nm TiO_2 wall thickness (T_D182) and being slightly larger than that of T_D91 and T_D273.

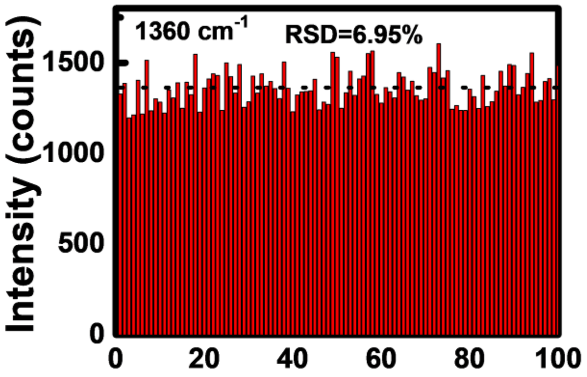

Figure S17. The SERS performances of Au NPs/SiO_2/Au RL/NIL resist _h316_T_D170 SERS chips with 1 nm thick SiO_2 layer on a 4-inch substrate. The SERS intensity distribution of 10^{-7} M R6G - decorated the chips from 100 random spots with a low RSD of 6.95 %.

Supplementary Table

Table S1. All the abbreviations with the corresponding indications.

Abbreviations	Indications
s(L_D)_T_D194 and y(L_D)_T_D194	Hollow square and parallel-wall (along *y*-direction) Au/SiO_2 structures with Au layers, a spacing of L_D, a thickness of T_D = 194 nm and a height of 228 nm for SiO_2 walls, respectively
s316_T_D9, s316_T_D90, s316_T_D135, s316_T_D194 and s316_T_D250	Hollow square Au/SiO_2 structures with a thickness of T_{PM} = 7 nm for Au layers, a spacing of L_D = 316 nm (i. e. $L_{PM\text{-}D}$ = 302 nm) and the thicknesses of T_D = 9, 90, 135, 194 and 250 nm and a height of 228 nm for SiO_2 walls, respectively
y316_T_D9, y316_T_D90, y316_T_D135, y316_T_D194 and y316_T_D250	Parallel-wall (along *y*-direction) Au/SiO_2 structures with a thickness of 7 nm for Au layers, a spacing of L_D = 316 nm (i. e. $L_{PM\text{-}D}$ = 302 nm) and and the thicknesses of T_D = 9, 90, 135, 194 and 250 nm and a height of 228 nm for SiO_2 walls, respectively
s316 and h316	Hollow square and hexagonal Au/SiO_2 structures with a thickness of 7 nm for Au layers, a spacing of L_D = 316 nm (i. e. $L_{PM\text{-}D}$ = 302 nm) and a height of 228 nm for SiO_2 walls, respectively
h316_T_D9, h316_T_D90, h316_T_D194, and h316_T_D250	Hollow hexagonal Au/SiO_2 structures with a thickness of 7 nm for Au layers, a spacing of L_D = 316 nm (i. e. $L_{PM\text{-}D}$ = 302 nm) and the thicknesses of T_D = 9, 90, 194 and 250 nm and a height of 228 nm for SiO_2 walls, respectively
h268_T_D164	Hollow hexagonal Ag-Au/SiO_2 structures with a thickness of 7 nm for Ag-Au layers, a spacing of L_D = 268 nm (i. e. $L_{PM\text{-}D}$ = 254 nm) and a thickness of T_D = 164 nm and a height of 228 nm for SiO_2 walls

Table S2. The calculative electric field (EF) enhancement $|E/E_0|^4$ for hollow hexagonal nanostructure at the incident light with *x*-polarization (Fig. 2c1) based on coupling effects of LSPRs and SPPs and electromagnetic waves interference.

**Hollow hexagonal structure at the incident light with *x*-polarization**	**Walls 1** (*y*-direction, i. e. The angles between them and *x*-direction are π/2.)	**Walls 2** (The angles between them and *x*-direction are π/6.)	**Walls 3** (The angle between them and *x*-direction are -π/6.)
**EF enhancement $\|E/E_0\|^4$**	$E_{LSPR\text{-}TM}{}^4 \cos^4(0)\ (I_{TM}+1)$ + $E_{LSPR\text{-}TE}{}^4\cos^4(\pi/2)$ • $(I_{TE}+1)$	$E_{LSPR\text{-}TM}{}^4\cos^4(\pi/3)$ • $(I_{TM}+1)$+ $E_{LSPR\text{-}TE}{}^4\cos^4(\pi/6)$ • $(I_{TE}+1)$	$E_{LSPR\text{-}TM}{}^4\cos^4(-\pi/3)$ • $(I_{TM}+1)$+ $E_{LSPR\text{-}TE}{}^4\cos^4(-\pi/6)$ • $(I_{TE}+1)$

Table S3. The SERS performance comparison between the reported Au-based SERS chips and our chips.

SERS Substrate	Probe Molecule	Detection Limit	Enhance-ment Factor	Signal Deviation	Refer-ences
Au nanoparticle /silica microsphere	Rhodamine 6G (R6G)	10^{-13} M	3.74×10^{10}	Less than 8%	[4]
Periodic porous Au nanoparticle array	R6G	10^{-9} M	1.4×10^{7}	Less than 6.6%	[5]
Au nanoparticles/ anodized aluminum oxide	R6G	10^{-10} M	2.4×10^{6}	/	[6]
Au petal-like shell structures	4-nitrobenzenethiol	10^{-15} M	5×10^{9}	/	[7]
Au nanoislands on Si wafer	R6G	10^{-9} M	10^{7}–10^{8}	/	[8]
Au nanorods	4-methylbenzenethiol	10^{-7} M	9.47×10^{8}	/	[9]
Au nanobipyramids/AAO nanoholes	4-aminothiophenol	/	1×10^{8} .	Less than 10%	[10]
Au RL/SiO_2 nanogrids	R6G	2.17×10^{-11} M	3.4×10^{8}	5.52%	[11]
Au nanoparticles/ SiO_2/Au RL/SiO_2 multilayer structure	R6G	9.5×10^{-14} M	8.9×10^{10}	5.13%	This work

Table S4. The optimized spacings and thicknesses of dielectric walls of hollow hexagonal structures according to the incident light wavelengths and optical properties of metals and dielectrics

Metal /Dielectric	λ_{Laser} (nm)	n_m	k_m	n_d	$\lambda_{SPP\ metal-air}$ (nm)	$\lambda_{SPP\ metal-dielectric}$ (nm)	$\lambda_{SPP\ metal-air} / n_d$ (nm)	L_D (nm)	T_D (nm)
Au/MgF_2	632.8	0.120	3.300	1.383[1]	603.0	415.4	436.0	316	208
Au/Si_3N_4	632.8	0.120	3.300	2.010[1]	603.0	249.6	300.0	316	250
Au/TiO_2	632.8	0.120	3.300	2.390[3]	603.0	182.4	252.3	316	182
Ag-Au/MgF_2	532	0.195	3.372	1.385[1]	508.0	350.1	366.8	268	175
Ag-Au/Si_3N_4	532	0.195	3.372	2.030[1]	508.0	209.1	250.2	268	210
Ag-Au/TiO_2	532	0.195	3.372	2.460[3]	508.0	147.6	206.5	268	148

References

[1] E. D. Palik, *Handbook of Optical Constants of Solids*, Academic Press, San Diego, CA, USA **1991**.

[2] E. D. Palik, *Handbook of Optical Constants of Solids*, Academic Press, San Diego, CA, USA **1985**.

[3] H. G. Tompkins, *J. Appl. Phys.* **1991**, *70*, 3876.

[4] W. Zhao, S. Xiao, Y. Zhang, D. Pan, J. Wen, X. Qian, D, Wang, H. Cao, W. He, M. Quan, Z. Yang, *Nanoscale* **2018**, *10*, 14220.

[5] G. Liua, K. Lia, Y. Zhanga, J. Dub, S. Ghafoorb, Y. Lu, *Appl. Surf. Sci.* **2020**, *527*, 146807.

[6] O. E. Cigarroa-Mayorga, S. Gallardo-Hernándezb, P. Talamás-Rohana, *Appl. Surf. Sci.* **2021**, *536*, 147674.

[7] Y. Zhang, Y. Gu, J. He, B. D. Thackray, J.Ye, *Nat Commun*. **2019**, *10*, 3905.

[8] Z. Fusco, R. Bo, Y. Wang, N. Motta, H. Chen, A. Tricoli, *J. Mater. Chem. C* **2019**, *7*, 6308.

[9] C. Zhuang, Y. Xu, N. Xu, J. Wen, H. Chen, S. Deng, *Sensors* **2018**, *18*, 3458.

[10] B. Lina, P. Kannanb, B. Qiua, Z. Lina, L. Guo, *Food Chem.* **2020**, *307*, 125528.

[11] Y. Tian, H. Wang, L. Yan, X. Zhang, A. Falak, Y. Guo, P. Chen, F. Dong, L. Sun, W. Chu, *Adv. Sci.* **2019**, *6*, 1900177.
